# The contrary intracellular and extracellular functions of PEDF in HCC development

**DOI:** 10.1038/s41419-019-1976-4

**Published:** 2019-10-03

**Authors:** Cen Li, Zhijian Huang, Liuqing Zhu, Xianhuan Yu, Tianxiao Gao, Juan Feng, Honghai Hong, Haofan Yin, Ti Zhou, Weiwei Qi, Zhonghan Yang, Chao Liu, Xia Yang, Guoquan Gao

**Affiliations:** 1 Program of Molecular Medicine, Affiliated Guangzhou Women and Children’s Hospital, Zhongshan School of Medicine, Sun Yat-sen University, Guangzhou, China; 2 Department of Biochemistry, Zhongshan School of Medicine, Sun Yat-sen University, Guangzhou, China; 3 Department of Pathology, School of Medicine, New York Medical College, Valhalla, New York USA; 4 Second Affiliated Hospital, Zhongshan School of Medicine, Sun Yat-sen University, Guangzhou, China; 5 Department of Medical Oncology, Sun Yat-sen University Cancer Center, Guangzhou, China; 6 Guangdong Engineering and Technology Research Center for Gene Manipulation and Biomacromolecular Products, Sun Yat-sen University, Guangzhou, China; 7 Guangdong Province Key Laboratory of Brain Function and Disease, Zhongshan School of Medicine, Sun Yat-sen University, Guangzhou, China

**Keywords:** Cancer metabolism, Tumour-suppressor proteins

## Abstract

Pigment epithelium-derived factor (PEDF), a classic angiogenic inhibitor, has been reported to function as a tumor suppression protein and to downregulate in many types of solid tumors. However, the expression level of PEDF and its role in hepatocellular carcinoma (HCC) are contradictory. The present study investigates the expression and different activities of secreted and intracellular PEDF during HCC development, as well as the underlying mechanism of PEDF on HCC lipid disorders. We found that PEDF had no association with patients’ prognosis, although PEDF was highly expressed and inhibited angiogenesis in HCC tumor tissues. The animal experiments indicated that full-length PEDF exhibited equalizing effects on tumor growth activation and tumor angiogenesis inhibition in the late stage of HCC progression. Importantly, the pro-tumor activity was mediated by the intracellular PEDF, which causes accumulation of free fatty acids (FFAs) in vivo and in vitro. Based on the correlation analysis of PEDF and lipid metabolic indexes in human HCC tissues, we demonstrated that the intracellular PEDF led to the accumulation of FFA and eventually promoted HCC cell growth by inhibiting the activation of AMPK via ubiquitin–proteasome-mediated degradation, which causes increased de novo fatty acid synthesis and decreased FFA oxidation. Our findings revealed why elevated PEDF did not improve the patients’ prognosis as the offsetting intracellular and extracellular activities. This study will lead to a comprehensive understanding of the diverse role of PEDF in HCC and provide a new selective strategy by supplement of extracellular PEDF and downregulation of intracellular PEDF for the prevention and treatment of liver cancer.

## Introduction

Hepatocellular carcinoma (HCC) is one of the most common malignant abdominal tumors and the third leading cause of cancer deaths worldwide^1^. The general incidence rises, while overall survival rate remains extremely low^2^. The curative treatment options of HCC, especially in the advanced stage, was limited^3,4^.

Pigment epithelium-derived factor (PEDF) belongs to the Serpin superfamily and is widely expressed in most organs, especially in the adipose tissue and liver^5,6^. It has been described as a natural angiogenesis inhibitor with multi-functional properties^5^. Previous studies have shown that PEDF expression is significantly downregulated during most cancer progression^7,8^. PEDF can potently prevent angiogenesis in different tumors including lung carcinoma, melanoma, and glioblastoma cancer, by either causing vascular endothelial cell death or inhibiting pro-angiogenic signals^7,8^. However, the role of PEDF in HCC development appeared to be controversial. Intravenous injection of PEDF-expressing human mesenchymal stem cells (hMSCs) on orthotopic nude mouse models of HCC caused a dramatic inhibition of primary tumor growth^9^. Similar results were found when applying exogenous PEDF on vascular endothelial cells (EC) and HCC xenografts^10–12^. These reports suggested that PEDF presented a classic inhibitory activity towards HCC angiogenesis and tumor progression^9–12^. Whereas more recent studies demonstrated that serum PEDF levels were higher in HCC patients than non-HCC patients, and PEDF expression was higher in human HCC tissues than adjacent non-tumor tissues^13,14^. Moreover, increased expressions of PEDF in HCC exert anti-apoptotic effects in tumor cells^13^ and promote tumor metastasis^14^. These opposite effects of PEDF triggered us to further investigate its function in HCC development.

Cancer cells reprogram their metabolic pathways to support their enhanced demands for proliferation and survival^15^. One of the best-known changes is the Warburg effect, which cancer cells mainly rely on glycolysis as an energy source even in normoxia^16^. Likewise, lipid metabolism is altered in rapidly proliferating cancer cells, such as activated de novo lipogenesis (DNL) pathways and high expression of monoacylglycerol lipase (MAGL)^17–19^, resulting in high levels of free fatty acids (FFAs) in tumor tissues. Elevated FFA levels support the malignancy of cancer cells by providing substrates for energy production^20^ and generating lipid-signaling molecules^21,22^. Recent studies have established PEDF as a novel yet crucial regulatory protein in lipid metabolism^23,24^ and our previous study showed intracellular PEDF promoted hepatic FFA release through modulating the key enzyme in Triglyceride (TG) catabolism, adipose TG lipase (ATGL)^25^. However, the specific role of PEDF in mediating FFA metabolism in HCC remains poorly defined.

In the present study, we unraveled the contrary intracellular and extracellular functions of PEDF in HCC development through its dual regulation on both tumor angiogenesis and FFA metabolism, which might clarify the early paradoxical results of PEDF on HCC progression and provide a potential therapeutic strategy for HCC.

## Materials and methods

### Reagents and antibodies

MTT ((4,5-dimethylthiazol-2-yl)-2,5-diphenyltetrazolium bromide), Oleic acid (OA), AICAR, Compound C, and C75 were from Sigma-Aldrich (St. Louis, MO, USA). Dulbecco’s modified Eagle’s medium (DMEM), fetal bovine serum (FBS), and 0.25%Trypsin-0.2%EDTA were from GIBCO (Gaithersburg, MD, USA). PrimeScript RT reagent Kit, Perfect Real Time kit, and SYBR Premix Ex Taq TM (Perfect Real Time) were from Takara Bio, Inc. (Japan). Bio-Rad DC protein assay kit was from Bio-Rad (Hercules, CA, USA). TRIzol reagent, Lipofectiamine 2000, puromycin, and Rabbit anti-CD31 primary antibody were from Thermo Fisher Scientific (Waltham, MA, USA). Anti-fatty acid synthase (FASN)/Acetyl-CoA carboxylase (ACC)/pACC/AMPK/pAMPK/ubiquitin/Rabbit normal IgG primary antibodies were from Cell Signaling Technology (Danvers, MA, USA). Anti-carnitine palmitoyltransferase-1a (CPT1α)/acyl-CoA dehydrogenase medium chain (ACADM) primary antibodies were from Proteintech (Chicago, IL, USA). Mouse anti-PEDF/β-actin/GAPDH primary antibodies and Chemikine PEDF ELISA Kit were from Millipore, Sigma (Burlington, MA, USA). Human Serpin F1/PEDF DuoSet ELISA Kit was from R&D Systems (Minneapolis, MN, USA). ECL detection kit was from Applygen Technologies, Inc. (Beijing, China). EnzyChromTM Free Fatty Acid Assay Kit and EnzyChromTM Triglyceride Assay Kit were from BioAssay Systems (Hayward, CA, USA). Attractene and HiPerFect transfection reagents were from Qiagen (Hilden, Germany). Cycloheximide (CHX; AC466) was purchased from Genview (Beijing, China); MG132 (S2619) was obtained from Selleck Chemicals (Houston, USA). RIPA Lysis Buffer was from Beyotime (Jiangsu, China). Protein A/G PLUS-Agarose (sc-2003) was purchased from Santa Cruz Biotechnology (Dallas, TX, USA).

### Patient samples and cell lines

Human HCC tissue samples were obtained from patients who underwent surgical treatment at the Sun Yat-sen Memorial Hospital (Guangzhou, China). The 74 HCC patients included 65 males and 9 females (mean age: 53 years, ranging from 26–82 years). All procedures were performed under consensus agreements and in accordance with the Chinese Ethical Review Committee. All tissue samples were fixed in 4% phosphate-buffered neutral formalin for at least 72 h and routinely embedded in paraffin.

HCC cell lines, Hep3B cells, and SK-Hep-1 cells were purchased from the Cell Bank of the Chinese Academy of Sciences (Shanghai, China). BEL-7402 cells were preserved in our laboratory. HepG2 cells were kindly provided by Professor Jun Li (ZhongShan School of Medicine, Sun Yat-sen University, China). All cell lines were maintained in DMEM supplemented with 10% FBS and were incubated at 37 °C under a humidified atmosphere with 5% CO_2_.

### TCGA data analysis

The RNASeq data and clinical data for HCC were obtained from The Cancer Genome Atlas (TCGA) databases (https://genome-cancer.ucsc.edu). For the association of PEDF expression with survival, patient vital status (dead and alive) was used as a surrogate endpoint and patients dichotomized by PEDF expression. Kaplan–Meier survival curves were constructed and the log-rank test was carried out using univariate analysis. Fifty pairs of HCC patients were collected and were used for analyzing gene expression difference of tumors and adjacent tissues.

### Generation of stable HepG2 or BEL-7402 cells

The lentivectors pLenti-EF1a-EGFP-F2A-Puro-CMV, pLenti-EF1a-EGFP-F2A-Puro-CMV- PEDF, and pLenti-EF1a-EGFP-F2A-Puro-CMV-mPEDF were purchased from OBiO Technology (Shanghai). HepG2 or BEL-7402 cells were infected with lentivectors carrying the (EGFP-) control, (EGFP-) PEDF, or (EGFP-) mPEDF transgene, as well as polybrene (6 μg/ml). After 16 h incubation, cells were cultured and passaged in regular culture condition (10% FBS + DMEM) for 2–5 days. Puromycin (0.8 μg/ml) was then added to the media for the screening of transfected cells for 7–10 days. The positively screened cells, termed the stable CON-, PEDF-, and mPEDF-HepG2 or BEL-7402 cells, were grown in 10% FBS + DMEM containing 0.8 μg/ml puromycin for further experiments.

### Tumor xenograft models

Male, 4-week-old athymic nude mice (BALB/c, nu/nu) were obtained from Experimental Animal Center of Sun Yat-sen University (Guangzhou, China; license number SCXK(YUE)2009–0011). Subcutaneous implants of the stable CON-, PEDF-, and mPEDF-HepG2 cells were performed by injecting 2 × 10^6^ cells into the right back flank of nude mice (*n* = 15 in each cell line). Tumor volume was monitored by caliper measurement every other day and was calculated according to the following formula: Volume = length × width^2^/2. Six mice were killed in each group in the second and sixth week. Tumor tissues were dissected, weighed, taken photos, and stored at −80 °C for further experiments.

All of the experiment procedures were reviewed and approved by the Institutional Animal Care and Use Committee of Sun Yat-sen University (IACUC SYSU, NO. 20061211005).

### Quantitative real-time PCR

Total RNA was extracted from HCC tissues or xenografts of nude mice or cultured cells according to the manufacturer’s instructions for TRIzol reagent. Total RNA (500 ng) was used for reverse transcription using PrimeScript RT reagent Kit and subjected to quantitative real-time PCR analysis (quantitative PCR, qPCR) using SYBR Premix Ex Taq TM and a Roche’s capillary-based Light Cycler 2.0 Systems. Target mRNA was determined using the comparative cycle threshold method of relative quantification. The calibrator sample was selected from adjacent non-tumor tissues or CON-HepG2 cell samples and β-actin was used as an internal control.

### Immunohistochemistry

Tumor tissues were fixed with 4% paraformaldehyde and cut into 5–10 μm paraffin-embedded sections. Tissue sections were deparaffinized and rehydrated using standard methods. After quenching endogenous peroxidase, slides were placed in 1 mM EDTA pH 7.5 and boiled for 15 min for antigen retrieval. After repeated washing and goat serum blocking, the slides were incubated with anti-PEDF or CD31 monoclonal antibody at 4 °C overnight. On the second day, the slides were treated with horseradish peroxidase-conjugated secondary antibody and the antigen–antibody complex was visualized by incubation with the DAB kit. Finally, all sections were counterstained with hematoxylin.

Tumor micro vessels density (MVD) was quantified using Weidner’s method. Briefly, the whole tumoral section was scanned at low power by microscope and identified the area of highest neovessel density, the so-called hot spot. Then, individual microvessels are counted at higher power (×200 field) in an adequate area. Any stained EC or clusters separate from adjacent vessels are counted as a single microvessel, even in the absence of vessel lumen. Every single count is expressed as the highest number of microvessels identified at the hot spot. Negative controls were incubated without the primary antibody.

### Western blot analysis

Tumor and adjacent non-tumor tissue samples from 74 HCC patients and 4 HCC xenografts randomly selected from each group at the second and sixth week were lysed with 1 × SDS buffer (tissue sample: buffer = 50 mg:1 ml) for total protein extraction. For cell samples, the cells were collected and lysed by 1 × SDS buffer after triple phosphate-buffered saline washing. Total lysate was then homogenized by sonication, boiled, and centrifuged at 12,000 r.p.m. at 4 °C. The supernatant was the total protein extraction. Protein concentration was determined using Bio-Rad DC protein assay kit. Equal amounts of total proteins (80 μg) were resolved by SDS-polyacrylamide gel electrophoresis and then electrophoretically transferred to polyvinylidene difluoride membranes. After blocking, the blots were incubated overnight with primary antibodies using dilutions suggested by the manufacturers. The same membrane was stripped and reprobed with an antibody specific to β-actin or glyceraldehyde 3-phosphate dehydrogenase (GADPH) as the loading control. For quantitative analysis, the bands were selected and quantified using Image J software and the data were normalized relative to β-actin or GADPH.

### Measurement of secreted PEDF

To quantify secreted PEDF, the serum from animal was centrifuged at 8000 r.p.m. for 10 min and the supernatant was collected and measured with a Human Serpin F1/PEDF DuoSet ELISA Kit according to the manufacturer’s instructions. For cell samples, cell culture medium was collected and extracellular PEDF content was determined by a Chemikine PEDF ELISA Kit, according to the manufacturer’s instructions.

### Cell viability assay

Cells were seeded in 24-well plates at a density of 2 × 10^4^ cells per well and maintained in the culture medium until they reached 60% to 70% confluence. Cells were starved without serum for 24/48/72 h. Cell viability was measured by MTT assay, according to the manufacturer’s protocol. Data represented absorbance and expressed as percentages of negative controls.

### Colony formation assay

Stable CON-, PEDF-, and mPEDF-HepG2 or BEL-7402 cells were seeded in 6-well plates at a density of 500 cells per well and were maintained in the culture medium. Every 3–4 days the medium was replaced with fresh medium containing puromycin. The assay was stopped after 2–3 weeks when the colonies were clearly visible even without looking under the microscope. The colonies were then stained with crystal violet and counted. Furthermore, the colony formation rates were calculated as the following formula: colony formation rate (%) = (the number of colonies/the number of cells seeded) × 100%.

### Measurement of triglycerides and free fatty acids

Lipid extraction from tissue samples was performed using a modified Folch method. Briefly, 50 mg tumor and adjacent non-tumor tissues were homogenized with 1 ml (chloroform:isopropanol:NP-40 = 7:11:0.1) extraction buffer on ice for 1 min and were sonicated for 30 s, then shook for 10 h and centrifuged for 20 min at 4000 r.p.m. The supernatant liquid was then transferred to a new tube and was evaporated under a vacuum at 50 °C for 2 h. After that, 200 µl ethanol was added to dissolve the lipid.

Lipid extraction from cell culture medium was conducted as described below. Different groups of HepG2 cells were treated with either bovine serum albumin (BSA) or 400 μM OA for 6 h, then washed and incubated with phenolred-free DMEM complexed with 1% BSA for an additional 12/24 h. The supernatant was collected and subjected to subsequent analyses. TG or FFA from tissue samples and cell culture medium was measured using the EnzyChromTM Triglyceride Assay Kit or EnzyChromTM Free Fatty Acid Assay Kit, according to the manufacturer’s instructions. Both TG and FFA were calculated from a standard curve for each assay and the data were normalized to total protein.

### Oil Red O staining

For lipid droplet staining, stable CON-, PEDF-, and mPEDF-HepG2 or BEL-7402 cells incubated with 400 μM OA for 6 h, or 10 μm cryostat sections from indicated HCC xenografts, were washed, fixed in 4% paraformaldehyde for 10 min, and rinsed with 60% isopropanol. The slides were then placed in the freshly prepared working Oil Red O solution for 10 min at room temperature and rinsed again with 60% isopropanol. After lightly stained nuclei with hematoxylin and washed with distilled water, the slides were covered with glycerine jelly that will harden after a few hours. Relative lipid content was quantified by using Image Pro Plus 6.0.

### Transient transfection and siRNA-mediated knockdown

Plasmids expressing mPEDF and PEDF were constructed and conserved by our lab. For transient transfection, HepG2 cells were transfected with EGFP-pcDNA3.1( + ), mPEDF-pcDNA3.1( + ), or PEDF-pcDNA3.1( + ) plasmids using Attractene reagent following the manufacturer’s instruction. After transfection for 24 h, the cells were then starved or collected for subsequent analysis.

For PEDF or FASN knockdown, a final concentration of 50 nM siRNAs (si-PEDF-1: 5′-GGAAAUUCCCGAUGAGAUCUUTT-3′; si-PEDF-2: 5′-CGAGUUCAUUCAUGACAU AGATT-3′; si-FASN: 5′-UCAUUUGAAUACAUCGAAGCCCACG-3′) were delivered to Hep3B cells or stable PEDF-HepG2 cells, respectively, using HiPerFect reagent. After transfection for 24/48 h, the cells were then starved or collected for subsequent analysis.

### Cycloheximide chase assay and protein half-life analysis

HepG2 cells were plated on six-well culture plates and transient transfection procedure was performed as described above. After transfection for 24 h, CHX were added to cells at a final concentration of 10 μg/ml to block new protein synthesis. Cells were then collected at the indicated time points for western blotting analysis. Intensity of the signals was quantified using Image J software and the data were plotted. For protein half-life analysis, data were log-transformed. A linear fit was performed to calculate the slope (constant, k) and the half-life (*T*_1/2_) using the equation *T*_1/2_ = ln(2)/k^26^.

### Co-immunoprecipitation

HepG2 cells were lysed in RIPA Lysis Buffer supplemented with protease and phosphatase inhibitors. Total cell lysates were then cleared by centrifugation and protein concentrations were measured by BCA assay. A small portion of the supernatant was saved as input control. Two micrograms of either anti-AMPKα or anti-pAMPKα antibody were added to 400 μg total protein and incubated at 4 °C for overnight. Then Protein A/G PLUS-Agarose (Santa Cruz) was added to incubate with the immunoprecipitants for an additional 4 h at 4 °C. Precipitated proteins were then washed three times with RIPA lysis buffer and boiled with 5× loading buffer, and immunoblotting was performed as previously described. Rabbit normal IgG was used as negative control.

### Statistical analysis

All data were expressed as mean ± SD. SPSS 13.0 software was used for one-way analysis of variance (ANOVA), Least Significant Difference (LSD) *t*-test, independent-samples *t*-test, paired-samples *t*-test, and log-rank test in statistical analyses. For more than two-group data, one-way ANOVA was used first to detect the difference among these groups, if the *p*-value was <0.05, and then multiple comparisons were performed using LSD *t*-test to detect the difference between any two groups. For the tumor and adjacent non-tumor tissues comparison, the paired-samples *t*-test method was used. For other two-group data, the independent samples *t*-test method was used. Kaplan–Meier survival curve was applied for the measurement of overall survival and log-rank test was used for comparison of survival rate in different groups. The correlation analyses were determined by the Pearson’s correlation test, whereas the association analyses were determined by the *χ*^2^-tests. All statistical tests were two-sided, with *p*-value <0.05 considered significant.

## Results

### PEDF is highly expressed in HCC patients without significant correlation with prognosis

**Fig. 1:**
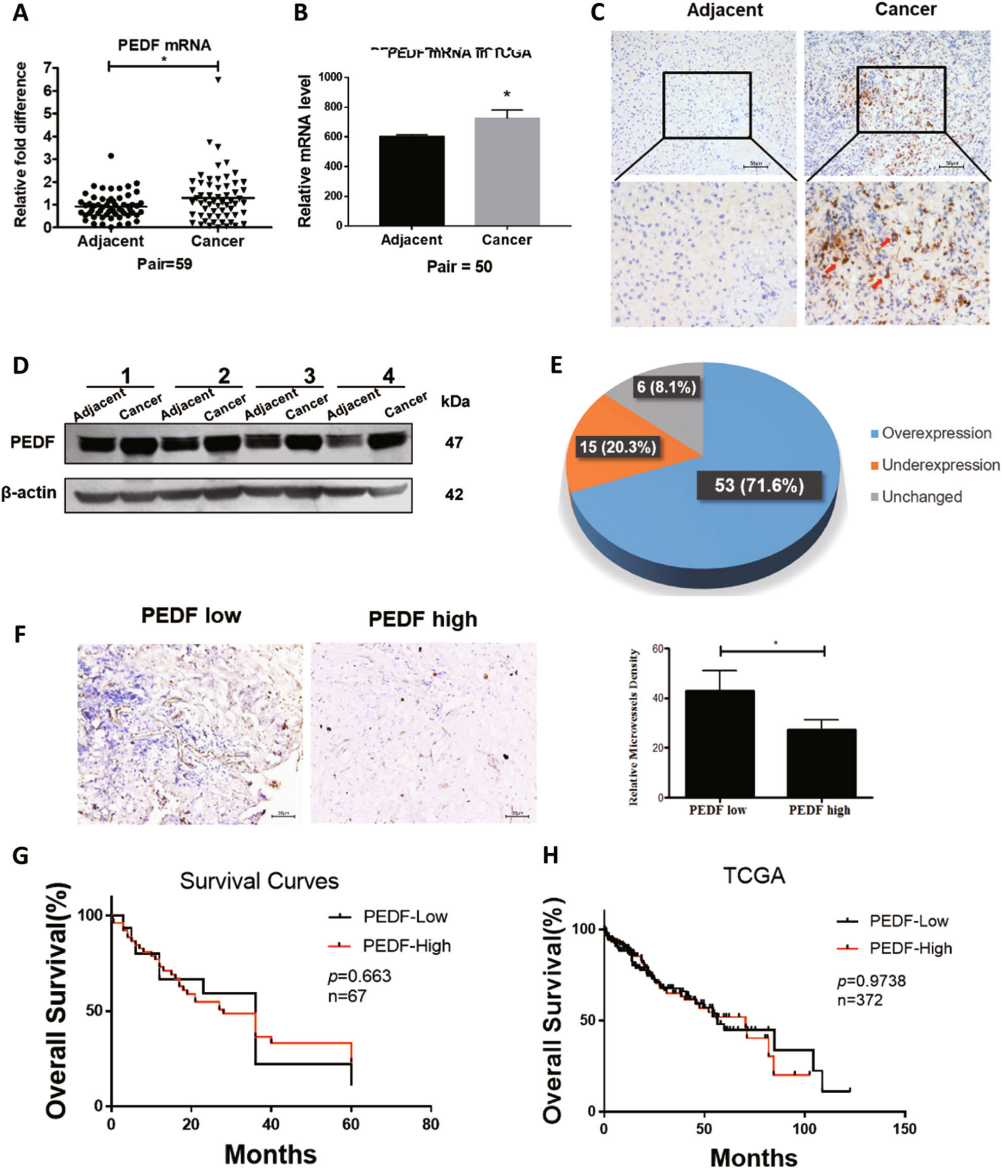
Expression levels and clinical significance of PEDF in HCC patients. Expression of PEDF mRNA level in HCC tissues (Cancer) and matched adjacent non-tumor tissues (Adjacent) were evaluated by **a** qPCR analysis (scatter plot + mean, *p < 0.05, *n* = 59) and **b** by analyzing the TCGA dataset (mean ± SD, *p < 0.05, *n* = 50). Expression of PEDF protein level in HCC tissues and matched adjacent non-tumor tissues were evaluated by **c** immunohistochemistry assay (representative images are shown, *n* = 10) and **d** western blot analysis (representative images are shown, *n* = 74). **e** The bands in Fig. 1d were quantified relative to β-actin and statistical analysis of PEDF protein expression in HCC samples were shown. **f** Immunostaining for CD31 and MVD assay were applied on PEDF low-expressed and high-expressed HCC tissues. Representative images are shown (mean ± SD, *p < 0.05, *n* = 3). **g** Overall survival analysis of HCC patients with PEDF expression (*n* = 67). **h** Overall survival analysis of HCC patients with PEDF expression in TCGA dataset (*n* = 372)

Previous studies reported commonly increased PEDF levels in HCC tissues compared with adjacent normal tissues^13,14^. To verify such expression pattern, HCC tissues and matched adjacent non-tumorous liver tissues were obtained from patients and were measured for expression levels of PEDF. As shown in Fig. 1a, PEDF mRNA expression was frequently higher in HCC tissues than in matched adjacent non-tumor tissues, in line with the results from TCGA dataset (Fig. 1b). Consistently, PEDF protein expression was remarkably increased in HCC tumor tissues, as found in both immunohistochemistry assay (Figs. 1c, 7 out of 10 pairs) and western blot analysis (Fig. 1d, e, 53 out of 74 pairs). Given the critical role of PEDF in tumor angiogenesis^7,8^, we applied MVD assay on HCC tissues and observed significantly reduced MVD in PEDF high-expressed HCC tissues compared with that in PEDF low-expressed HCC tissues (Fig. 1f). These data support previous findings of higher PEDF expression levels in HCC tissues and suggest its classic anti-angiogenic function in inhibiting neovascularization. However, the expression levels of PEDF showed no significant correlation with the overall survival time in HCC patients (Fig. 1g) nor with most clinicopathological features (Supplementary Table. 1). In addition, TCGA dataset analysis also revealed no evident correlation between PEDF expression and HCC prognosis (Fig. 1h). These results contradicted its inhibitory effect on tumor angiogenesis and led us to speculate other activities of PEDF in HCC development.

### Dual effects of PEDF overexpression on different stages of HCC development in vivo

**Fig. 2:**
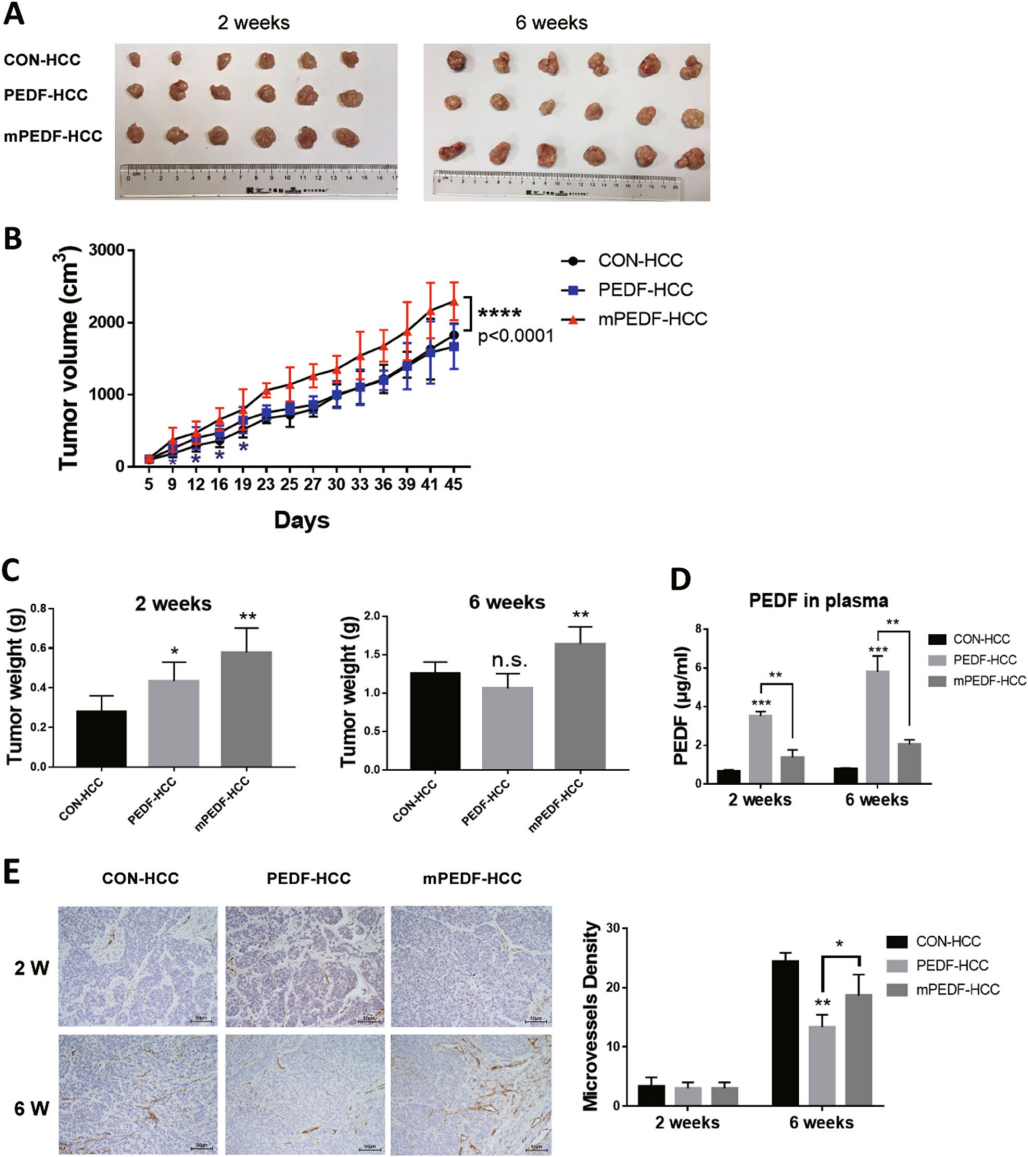
Dual effects of PEDF on different stages of tumor development in HCC xenograft models. Heterotopic HCC xenograft mouse models were performed using CON-, PEDF-, and mPEDF-HepG2 cells. Tumors were collected in the second and sixth week (*n* = 6). Representative images of tumors (**a**), the tumor growth curve (**b**), tumor weight (**c**), and secreted PEDF levels in the plasma (**d**) of CON-, PEDF-, and mPEDF-HCC groups at different time points were shown. **e** Immunostaining for CD31 and MVD assay were applied on indicated tumor xenografts collected in the second (2 W) and sixth week (6 W) (*n* = 3). All data are presented as mean ± SD and *p < 0.05, **p < 0.01, ***p < 0.001, and ****p < 0.0001, respectively

To fully characterize the role of PEDF in HCC development, we stably expressed vector (CON), full-length PEDF (PEDF), and signal-peptide-deleted PEDF retaining its expression in cytoplasm (mPEDF) in HepG2 cells with lentiviral infection. Heterotopic tumor xenograft mouse models were established using these stable HepG2 cells and tumor growths were monitored accordingly. As shown in Fig. 2a–c, tumor growths in both PEDF-HCC and mPEDF-HCC groups were significantly higher than those in the CON-HCC group in the first 2 weeks. After that, tumors in PEDF-HCC group started to grow slower and by the end of the sixth week they were slightly outgrown by those in the CON-HCC group (*p* > 0.05). The surprisingly declined tumor growth rate exhibited at the later stage in PEDF-HCC group is consistent with our clinical observations that high expression of PEDF does not correlate with HCC patient outcomes. On the other hand, tumors in the mPEDF-HCC group presented a steady, increased growth rate and dramatically surpassed tumors in the other two groups in 6 weeks’ time, indicating that intracellular PEDF could continuously promote tumor growth without secretory activity.

Secreted PEDF levels in the plasma of each group were validated by enzyme-linked immunosorbent assay (ELISA). While PEDF-HCC group exhibited significantly higher plasma PEDF levels throughout the experiment, the increase in plasma PEDF levels of the mPEDF-HCC group was subtle (Fig. 2d). Furthermore, as shown in MVD assay, very few neo-vessels appeared within tumors from all three groups in the second week. However, more vessels started to emerge as the tumors grew larger. By the end of sixth week, PEDF overexpression strongly restrained the number of neo-vessels in HCC xenografts, whereas mPEDF overexpression had limited inhibitory effect on tumor angiogenesis (Fig. 2e).

Taken together, these data indicate the equalizing effects of full-length PEDF on HCC development in vivo: intracellular PEDF might display a pro-tumoral activity that accelerates tumor growth, whereas in the late stage, secreted PEDF might act as a classic anti-angiogenic factor that suppresses angiogenesis in tumor progression.

### Intracellular PEDF promotes HCC cell proliferation in vitro

**Fig. 3:**
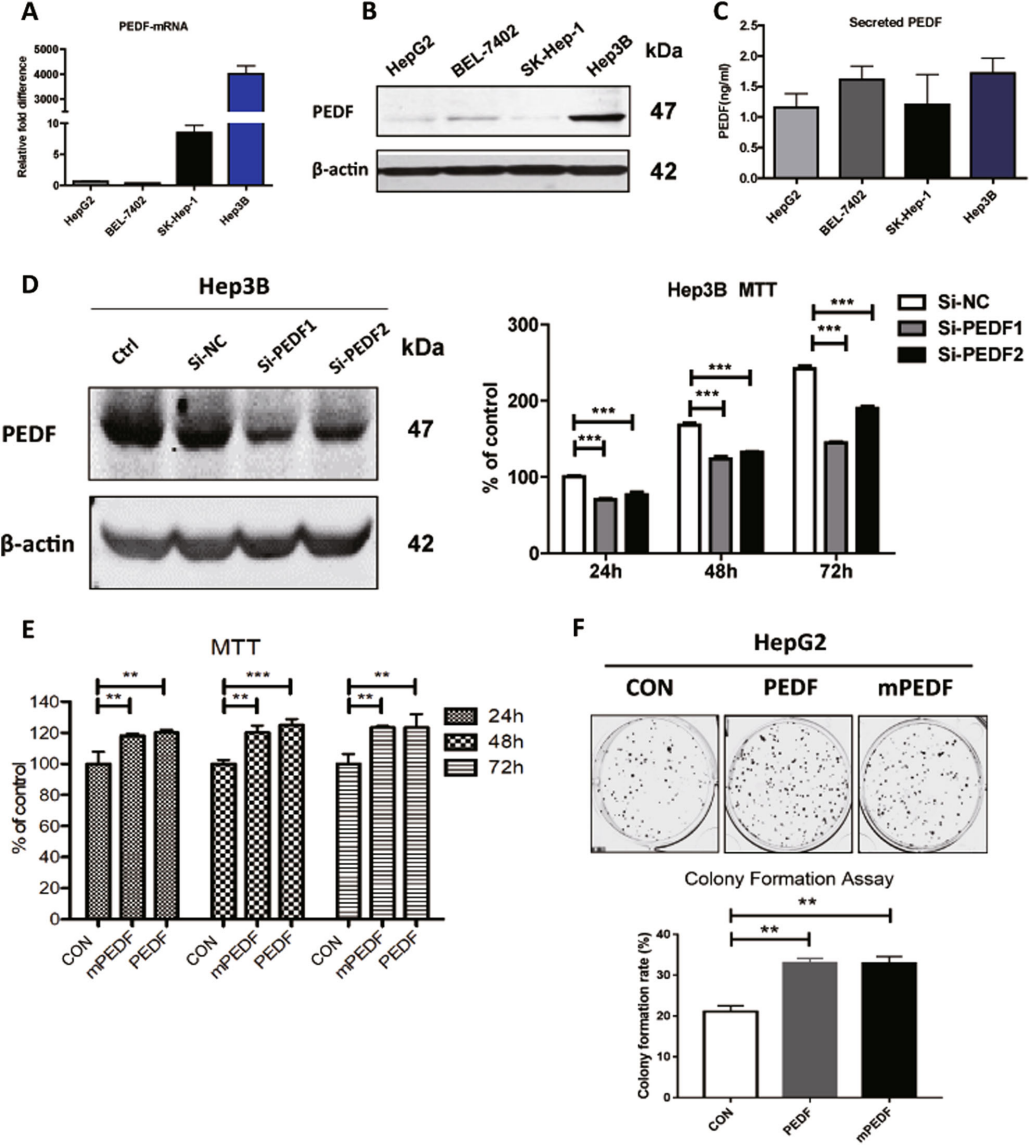
Intracellular PEDF promotes HCC cell proliferation in vitro. Expression of PEDF mRNA levels (**a**), protein levels (**b**), and secreted PEDF levels (**c**) in different HCC cell lines were evaluated by qPCR, western blot analysis, and ELISA assay, respectively. **d** Hep3B cells were transfected with reagent only (Ctrl) or a scrambled siRNA (si-NC), or two different siRNAs targeted PEDF (si-PEDF1 and si-PEDF2) for 24 h. Cells were then starved for an additional 24/48/72 h and MTT assay was performed to show the percentage of viable cells (*n* = 6). **e** HepG2 cells were first transfected with indicated plasmids for 24 h and then starved for 24/48/72 h. The viable cells were quantified by MTT assay (*n* = 6). **f** Stable CON-, PEDF-, and mPEDF-HepG2 cells were seeded and colony formation assay was performed as described in “Materials and Methods.” Representative images and colony formation rates are shown. All data are presented as mean ± SD and *p < 0.05, **p < 0.01, and ***p < 0.001, respectively

To investigate how PEDF mediates HCC development in vitro, we assessed the endogenous expression levels of PEDF in different HCC cell lines. As shown in Fig. 3a–c, HepG2 cells consistently expressed low levels of PEDF, whereas Hep3B cells had the strongest basal levels of PEDF expression across the board. PEDF expression was then transiently knocked down in Hep3B cells and significantly fewer viable cells were detected in PEDF knockdown groups than in the control group, suggesting reduced PEDF levels could impair HCC cell growth and reproduction (Fig. 3d). Given that intracellular PEDF promotes tumor growth in vivo (Fig. 2), HepG2 cells were transiently transfected with vector, PEDF, or mPEDF plasmids (Supplementary Fig. 1A-C) and cell viability were measured. Compared with the control group, overexpression of either PEDF or mPEDF caused remarkable increase in cell survival (Fig. 3e). Similarly, colony formation assay was performed using stable HepG2 cells and evidently more colonies were found in PEDF-HepG2 and mPEDF-HepG2 groups (Fig. 3f). Such stimulations on cell growth and division by PEDF and mPEDF were also observed in stable BEL-7402 cells (Supplementary Fig. 2A-D), implying the feature was not restricted to HepG2 cells. Together, these data support the notion that intracellular PEDF promotes HCC cell proliferation in vitro.

### The association between PEDF expression and lipid metabolism in HCC patients

**Fig. 4:**
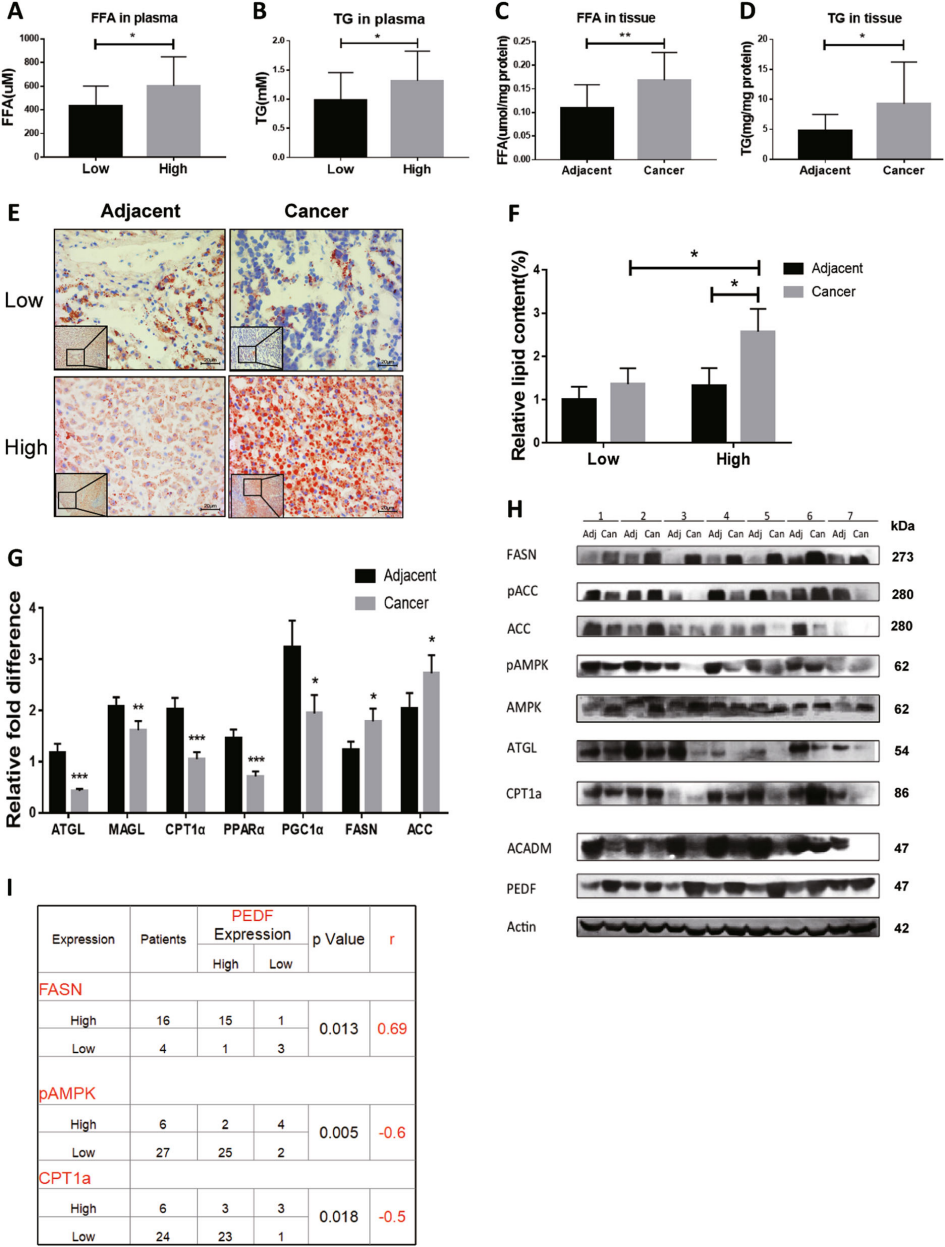
The association between PEDF expression and lipid metabolism in HCC patients. Plasma FFA levels (**a**, *n* = 33) and plasma TG levels (**b**, *n* = 46) were evaluated in HCC patients (Low, PEDF low-expressed HCC patients; High, PEDF high-expressed HCC patients). FFA levels and TG levels (**c**, **d**, *n* = 14) in PEDF high-expressed HCC tissues (Cancer) and matched adjacent non-tumor tissues (Adjacent) were evaluated. **e** Oil Red O staining was performed to determine lipid droplets in HCC tissues and matched adjacent non-tumor tissues. Representative images are shown (Low, PEDF low-expressed tissues; High, PEDF high-expressed tissues) and **f** relative lipid content was quantified using Image Pro Plus 6.0 (*n* = 3). **g** mRNA expression levels of lipid metabolic regulators in HCC tissues and matched adjacent non-tumor tissues were evaluated by qPCR analysis (ATGL: *n* = 45, MAGL: *n* = 43, CPT1α and PPARα: *n* = 30, PGC1α: *n* = 31, FASN and ACC-1: *n* = 23). **h** Protein expression levels of lipid metabolic enzymes in HCC tissues (Can) and matched adjacent non-tumor tissues (Adj) were evaluated by western blot analysis. **i** Correlation analysis between PEDF protein expression and FASN protein levels (*n* = 20), pAMPK protein levels (*n* = 33), and CPT1α protein levels (*n* = 30) in HCC patients. All data are presented as mean ± SD and *p < 0.05, **p < 0.01, and ***p < 0.001, respectively

Given the recent reports of PEDF as a critical modulatory molecule in hepatic lipid homeostasis^23–25,27^ and dysregulated lipid levels as a hallmark in cancer progression^17,19^, we tried to determine the role of PEDF on lipid metabolism in HCC patients. First, patients were divided into two groups according to their PEDF expression levels in HCC tissues and plasma FFA and TG levels were compared between groups. As shown in Fig. 4a, b, HCC patients with high expression of PEDF presented significantly higher levels of plasma FFA and plasma TG than those with low PEDF expression. Next, focusing on PEDF high-expressed HCC patients, we found markedly elevated FFA and TG levels in HCC tissues compared with matched adjacent non-tumor tissues (Fig. 4c, d). Lastly, HCC patients with low PEDF expression displayed globally low lipid contents, whereas patients with high PEDF expression exhibited strong lipid accumulation in HCC tissues (Fig. 4e, f).

To further elucidate how PEDF regulates lipid metabolism in HCC patients, we surveyed the expression of genes involved in lipid biosynthesis and lipid degradation. As shown in Fig. 4g, h, we observed impaired TG hydrolysis and FFA oxidation pathways in HCC tissues, manifested as significantly reduced expressions of ATGL, MAGL, CPT1α, peroxisome proliferator-activated receptor-α (PPARα), PPAR-γ coactivator 1α, ACADM, and 5′-AMP-activated protein kinase (AMPK), the key regulator in FFA metabolism. Meanwhile, expressions of FASN and ACC-1 are effectively induced in HCC tissues, suggesting enhanced DNL pathway. Statistical analysis of protein levels in HCC tissues revealed that PEDF expression was positively associated with the expression of FASN, the key enzyme in DNL pathway, whereas it was negatively associated with the activation of AMPK (pAMPK) and the expression of CPT1α, the key enzyme in FFA oxidation pathway (Fig. 4i). These data strongly implicate that PEDF might play a crucial part in FFA metabolism that contributes to HCC development.

### PEDF induces FFA and TG accumulation in HCC cells in vitro and in vivo

**Fig. 5:**
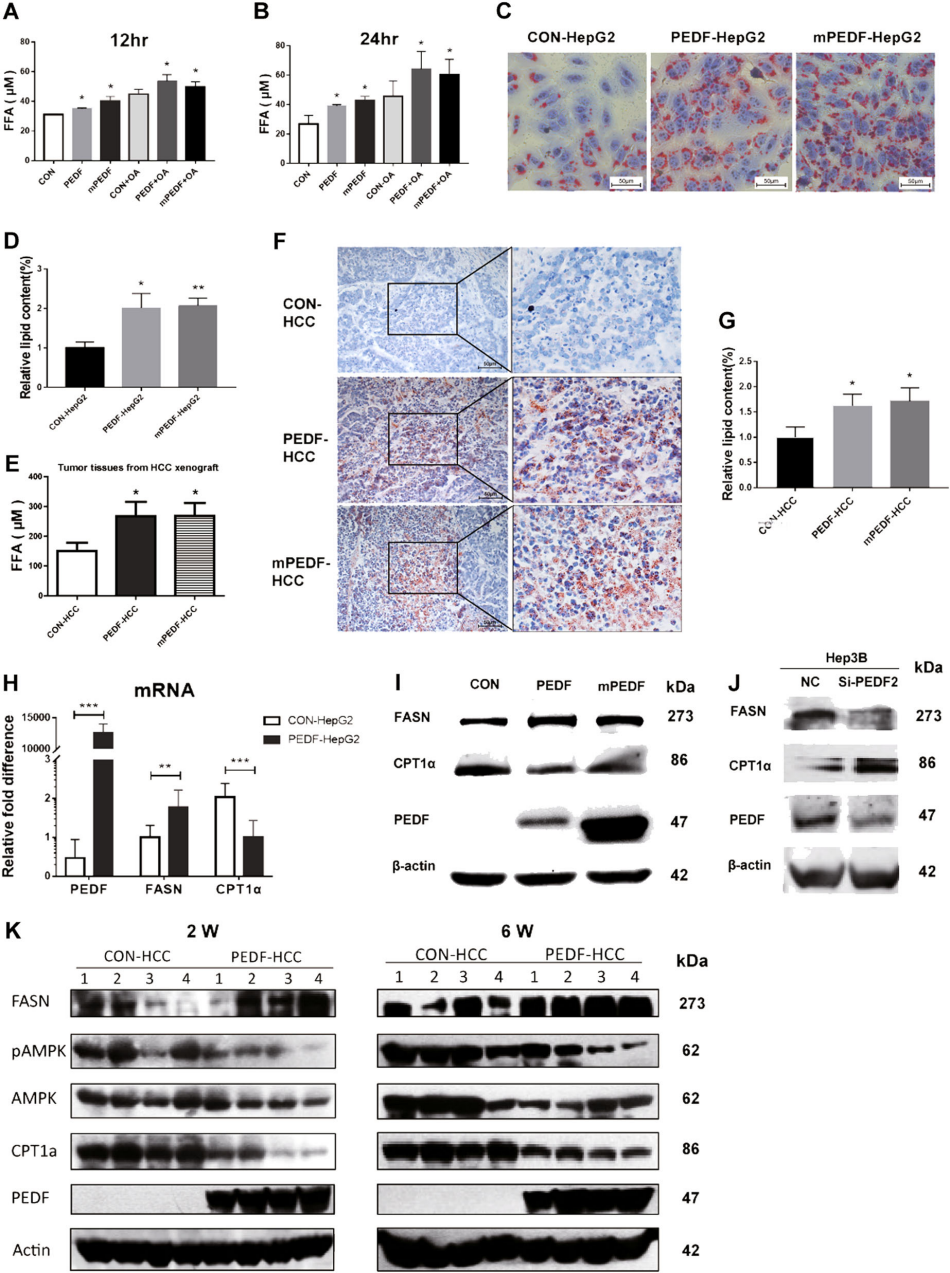
PEDF induces FFA and TG accumulation in vitro and in vivo. **a**, **b** Stable CON-, PEDF-, and mPEDF-HepG2 cells were treated with either BSA or 400 μM OA for 6 h, then switched to DMEM complexed with BSA for indicated time periods. FFA levels in the media were evaluated as described in “Materials and Methods” (*n* = 3). **c** Lipid droplets in indicated stable HepG2 cells were treated with 400 μM OA for 6 h and then stained with Oil Red O. Representative images and **d** quantification of lipid content (*n* = 3) are shown. **e** FFA levels in CON-, PEDF-, and mPEDF-HCC xenografts on the sixth week were evaluated (*n* = 6). **f** Lipid droplets in indicated HCC xenografts on the sixth week were stained with Oil Red O. Representative images and **g** quantification of lipid content (*n* = 3) are shown. **h** HepG2 cells were transfected with control (CON-HepG2) or PEDF (PEDF-HepG2) plasmids for 24 h and then collected and subjected to qPCR analysis (*n* = 3). **i** HepG2 cells were transfected with indicated plasmids for 24 h and then starved for 24 h. Cells were collected for western blot analysis. **j** Hep3B cells were transfected with a scrambled siRNA (NC) or an siRNA targeted PEDF (si-PEDF2) for 48 h. Cells were then collected for western blot analysis. **k** CON-HCC and PEDF-HCC xenografts were collected in the second week (2 W) and the sixth week (6 W). Four xenografts randomly selected from each group were subjected to total protein extraction and subsequent western blot analysis. All data are presented as mean ± SD and *p < 0.05, **p < 0.01, and ***p < 0.001, respectively

To verify the regulatory effect of PEDF on FFA metabolism in HCC cells, culture medium of stable HepG2 cells were collected and measured for FFA levels. As shown in Fig. 5a, b, both PEDF-HepG2 and mPEDF-HepG2 cells demonstrated significantly higher FFA levels than CON-HepG2 cells, especially in groups with OA treatment. Consistently, Oil Red O staining showed that PEDF-HepG2 and mPEDF-HepG2 cells had evidently more and larger lipid droplets in the cytoplasm than CON-HepG2 cells (Fig. 5c, d), suggesting increased lipid accumulation in these two cells. Similar results were found on HCC xenograft mouse models. Compared with CON-HCC group, remarkably elevated FFA levels were found in PEDF-HCC and mPEDF-HCC xenografts (Fig. 5e), coupled with considerably higher levels of TG accumulation in these two groups (Fig. 5f, g). These data illustrate that PEDF promotes FFA and TG accumulation in HCC cells, both in vitro and in vivo.

To further explore the role of PEDF in lipid metabolic pathways in HCC cells, HepG2 cells were transfected with indicated plasmids and assessed for mRNA and protein expressions. As shown in Fig. 5h, i, overexpression of both PEDF and mPEDF could significantly upregulate FASN expressions, whereas downregulating CPT1α expressions. Besides, silencing endogenous PEDF expression in Hep3B cells efficiently reduced FASN protein expression, whereas inducing CPT1α protein expression (Fig. 5j). Moreover, aforementioned HCC xenografts were collected and analyzed in the second and sixth week. In comparison with the CON-HCC group, tumors in PEDF-HCC group exhibited persistent increased FASN levels, as well as decreased CPT1α and pAMPK levels (Fig. 5k). Together with our clinical findings (Fig. 4), these data indicate that PEDF might activate FFA biosynthesis pathway in HCC cells, cause elevated FFA and TG levels, and therefore promote HCC progression.

### Intracellular PEDF induces FFA accumulation via AMPK activity and further promotes HCC cell proliferation

**Fig. 6:**
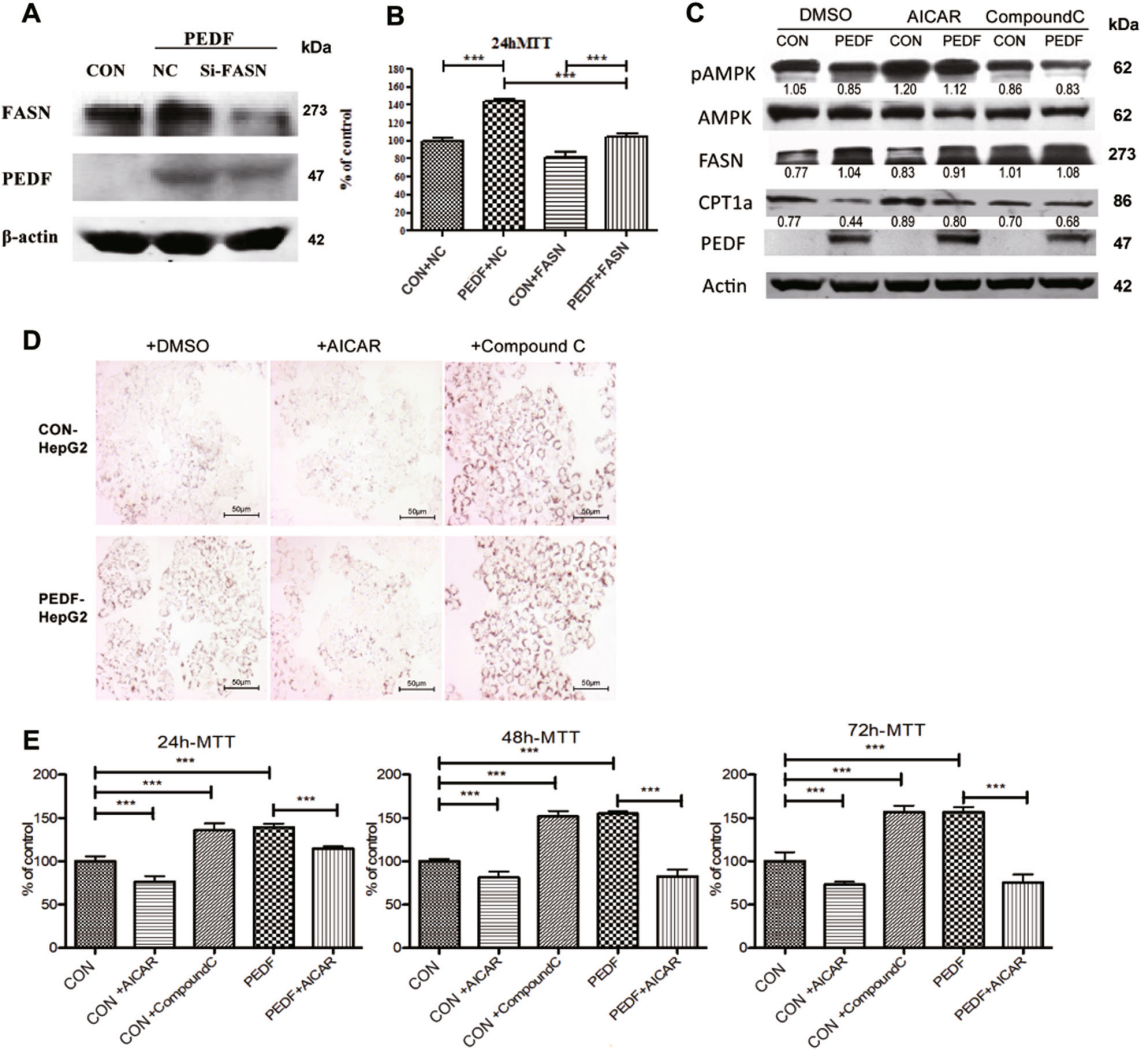
Intracellular PEDF mediates FFA metabolism via AMPK and further promotes HCC cell proliferation. **a** Stable PEDF-HepG2 cells (PEDF) were transfected with a scrambled siRNA (NC) or an siRNA-targeted FASN (si-FASN) for 48 h. Cells were then collected for western blot analysis (CON, CON-HepG2 cells). **b** CON- and PEDF- HepG2 cells were transfected with indicated siRNAs for 24 h and then starved for additional 24 h. The viable cells were quantified by MTT assay (mean ± SD, ***p < 0.001, *n* = 6). **c** CON- and PEDF- HepG2 cells were treated with either DMSO or AMPK agonist AICAR (250 μM), or AMPK inhibitor Compound C (1 μM), for 24 h. Cells were then collected for western blot analysis. **d** CON- and PEDF- HepG2 cells were first treated with 400 μM OA for 6 h then switched to DMSO, AICAR (250 μM), or Compound C (1 μM) treatment for additional 12 h. Cells were then stained by Oil Red O to determine lipid droplets. Representative images are shown. **e** CON- and PEDF-HepG2 cells were treated by AICAR (250 μM) or Compound C (1 μM) for 24/48/72 h. The viable cells were quantified by MTT assay (mean ± SD, ***p < 0.001, *n* = 6)

Our studies found intracellular PEDF effectively enhance HCC cell proliferation (Fig. 2, 3). Our further studies showed PEDF expression in HCC positively associated with FFA accumulation in vivo and in vitro (Fig. 4, 5). We then tried to clarify the significance of PEDF modulatory activity towards FFA in HCC cell proliferation. As shown in Fig. 6a, b, depletion of FASN in stable PEDF-HepG2 cells partially reversed the accelerated cell growth caused by PEDF overexpression. Similar results were also found when using the specific inhibitor of FASN, C75, on these stable HepG2 cells (Supplementary Fig. 3).

In search of the underlying mechanism how PEDF regulates FFA metabolic pathways, AMPK, the central controller in hepatic lipid metabolism^28^, caught our attention. Given the inverse relationship between PEDF expression and activation of AMPK (pAMPK) in HCC patients (Fig. 4), and constant decreased pAMPK expression in PEDF-HCC xenografts (Fig. 5k), we reasoned that PEDF could mediate the activity of AMPK. To test this hypothesis, we first treated stable HepG2 cells with AMPK stimulator, AICAR, and checked whether it could reverse the effect of PEDF on FFA metabolism. As shown in Fig. 6c, d, AICAR efficiently attenuated the elevated lipogenesis pathway and TG contents in PEDF-HepG2 cells. Furthermore, AICAR dramatically suppressed cell survival in both stable HepG2 cells, to the extent that completely diminished the increase in cell growth induced by PEDF (Fig. 6e). On the other hand, the AMPK inhibitor, Compound C displayed a synergetic effect on lipid metabolism and cell proliferation with PEDF (Fig. 6c–e). These data bespoke the activity of AMPK might be the main target of PEDF regulation on FFA metabolism.

### PEDF suppresses AMPK level and activity through ubiquitin–proteasome-mediated protein degradation

**Fig. 7:**
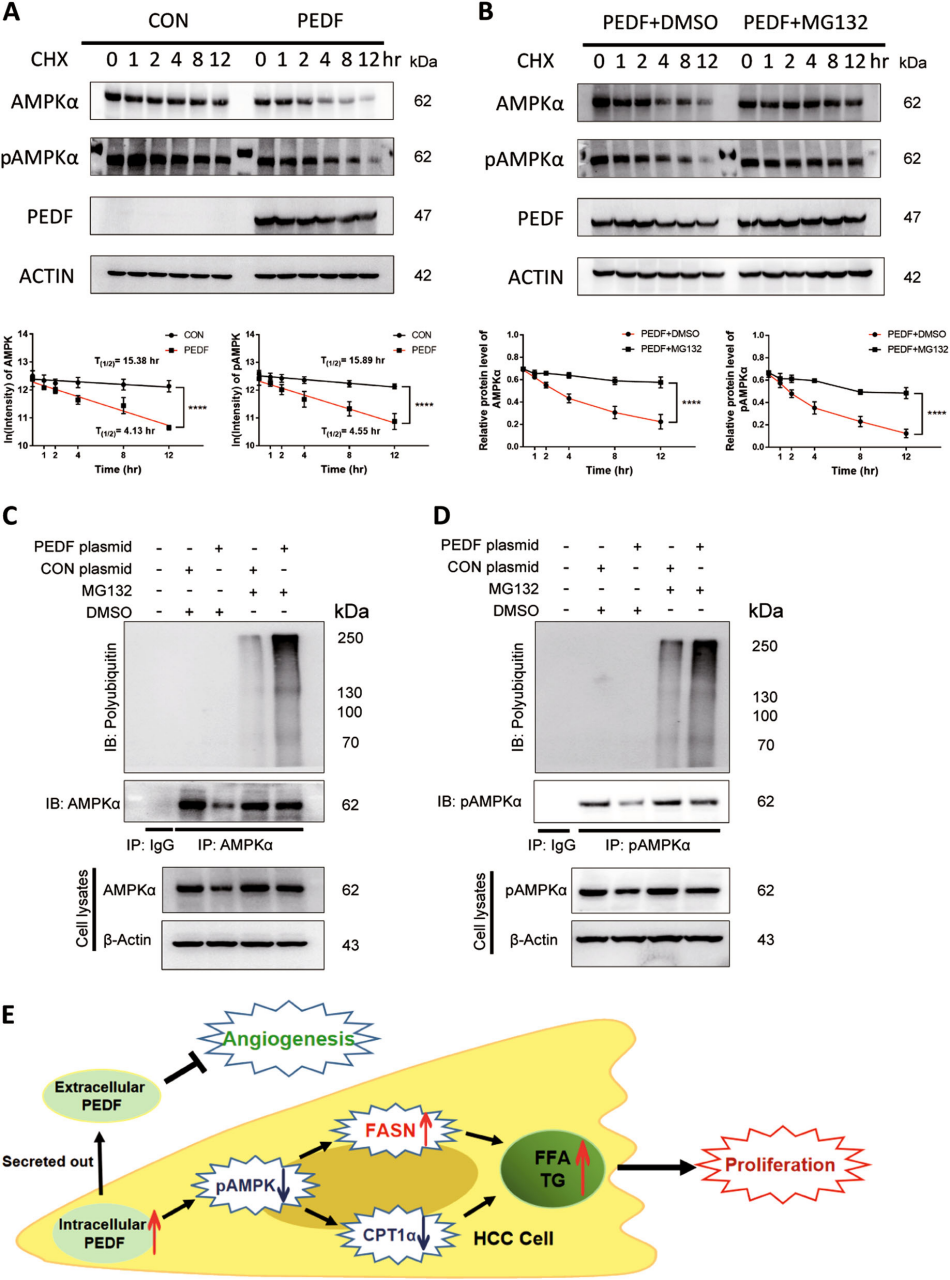
PEDF induces AMPK degradation through the ubiquitin–proteasome system. **a**, **b** Cycloheximide chase assay of AMPK and pAMPK. **a** HepG2 cells transiently transfected with vector (CON) or PEDF (PEDF) plasmids were treated with cycloheximide (CHX, 10 μg/ml) for indicated time points and then collected for western blotting analysis. AMPK and pAMPK protein signals were quantified, the data were log-transformed, and the protein half-lives were calculated as described in “Materials and Methods” (mean ± SD, ****p < 0.0001, *n* = 3). **b** HepG2 cells transfected with PEDF (PEDF) plasmid were treated with DMSO or MG132 (10 μmol/l) in the presence of CHX (10 μg/ml) for indicated time points and then collected for western blotting analysis. AMPK and pAMPK protein signals were quantified and the data were plotted (mean ± SD, ****p < 0.0001, *n* = 3). **c**, **d** HepG2 cells transiently transfected with CON or PEDF plasmids were treated with DMSO or MG132 (10 μmol/L) for 12 h, and then immunoprecipitated with an **c** AMPK antibody or **d** pAMPK antibody. The ubiquitination level of AMPK or pAMPK was determined using an anti-ubiquitin antibody. IB, immunoblotting; IP, immunoprecipitation. **e** Overview of the dual regulation of PEDF on HCC tumor development. On one hand, intracellular PEDF inhibits the activation of AMPK, which activates lipogenesis pathway by increasing FASN expression and suppresses FFA oxidation pathway by decreasing CPT1α expression, leads to elevated FFA levels, and eventually promotes HCC cell proliferation. On the other hand, secreted PEDF performs as a classic anti-angiogenic factor that inhibits tumor angiogenesis in HCC progression. Taken together, PEDF might present dual regulatory effects on HCC development

To investigate how PEDF modulates the activation of AMPK, we first measured mRNA expressions of two AMPKα isoforms in stable HepG2 cells and found no significant changes (Supplementary Fig. 4A-C), suggesting AMPK mRNA level may not contribute to the repressed activation. AMPK activity was reported to be regulated by posttranslational modifications besides phosphorylation, specifically ubiquitination^29,30^. To assess the effect of PEDF on AMPK protein stability, CHX chase assay was performed. We observed dramatic drops in protein half-lives of both AMPK and pAMPK in PEDF-overexpressed groups compared with control groups (Fig. 7a). While applying a specific proteasome inhibitor, MG132, the suppressive effects of PEDF on both AMPK and pAMPK were mostly eliminated (Fig. 7b), indicating that AMPK level and activity were indeed inhibited by PEDF through the proteasome-mediated degradation pathway. Further, we examined whether AMPK is ubiquitinated and whether PEDF can affect its ubiquitination. HepG2 cells were transfected with indicated plasmids with or without MG132 treatment, followed by immunoprecipitation with an AMPK antibody. As shown in Fig. 7c, endogenously ubiquitinated AMPK was merely detectable with proteasome inhibition, whereas the polyubiquitination level was remarkably augmented when treatment coupled with PEDF overexpression. Similar trends were found using the ubiquitination assay with a pAMPK antibody, that PEDF with MG132 strongly promoted pAMPK polyubiquitination (Fig. 7d). Given that ubiquitination may facilitate a negative impact on AMPK activation^29–31^, these data implicate that PEDF could block AMPK activity and quantity through the ubiquitin–proteasome system (UPS).

## Discussion

Over the past few decades, PEDF has been described as a potent secretory angiogenesis inhibitor that blocks tumor progression^5^. Downregulation of PEDF expression is linked to increased metastases and poor prognosis in many human cancer types^5,8,32^. Although exogenous PEDF has been reported to inhibit HCC tumor growth through restraining MVD and vascular endothelial growth factor (VEGF) expression^9–12,33^, recent studies found high expressions of PEDF in HCC patients, and that PEDF could inhibit HCC cell apoptosis^13^ and promote HCC metastasis^14^. The present study proposes a possible explanation for the opposing roles of PEDF in HCC development by revealing the dual regulatory functions of PEDF during tumor progression. Other than its traditional extracellular activity of inhibiting angiogenesis, we unravel the novel function of intracellular PEDF in inducing FFA accumulation further promoting HCC cell proliferation. Moreover, intracellular PEDF exerts the effect on FFA metabolism mainly through repressing the activation of AMPK via ubiquitin–proteasome-mediated protein degradation. These new findings clarify previous controversial reports and provide mechanistic insights on exploring therapeutic potential of PEDF in HCC.

“Angiogenic switch” happens when a tumor reaches a certain size and demands more nutrients for expansion^34^. In most cancer progression, PEDF can effectively suppress tumor angiogenesis through downregulating VEGF expression^35^ and inducing endothelial cell apoptosis^5^. Using bioengineered hMSCs, researchers concentrated high-level secreted PEDF towards HCC and significantly retarded tumor growth and neo-angiogenesis^9^. Consistent with these findings, we observed much fewer MVD in PEDF high-expressed tumor tissues than PEDF low-expressed tumor tissues in HCC patients (Fig. 1f), and blunted tumor growth rate in PEDF-HCC xenografts due to evidently decreased MVD at the late stage of tumor progression (Fig. 2e). Further, conditioned media derived from PEDF-HepG2 cells could dramatically reduce vascular endothelial cell survival (Supplementary Fig. 1D), indicating the classic anti-angiogenic function of secreted PEDF both in vivo and in vitro.

However, conflictive with the inhibitory effect, we found higher PEDF expression levels in HCC tissues than matched adjacent non-tumor tissues (Fig. 1a–e), and no correlation between PEDF expression and HCC patient outcomes (Fig. 1g, h). These data are in accordance with recent findings of elevated PEDF levels in serum and tumor tissues of HCC patients^13,33^, which may give rise to HCC growth and metastasis^13,14^. Our animal studies further demonstrated that PEDF overexpression could remarkably induce tumor growth at the early stage, whereas mildly reduce tumor growth at the late stage (Fig. 2a–c). The present study elucidates these discrepant activities by establishing that PEDF performs different functions based on its distributions in HCC cells. Using strategy-sequestered PEDF expression inside the cell (mPEDF), our further experiments revealed that although both mPEDF and PEDF possessed similar capability in enhancing HCC cell growth in vitro (Fig. 3 and Supplementary Fig. 2), only mPEDF overexpression could persistently accelerate tumor growth in vivo (Fig. 2). Therefore, our study uncovered, for the first time, the significant role of intracellular PEDF in promoting HCC cell proliferation. Together, these data suggest that PEDF could cause equalizing effects on HCC progression through offsetting extracellular and intracellular activities, which might contribute to its lack of association with HCC prognosis.

Dysregulated lipid metabolism underlies HCC pathogenesis, manifested as aberrantly activated DNL pathway, facilitating drastic increases in FFA levels and cell survival in tumors^36–38^. When analyzing the lipid metabolic indexes in HCC patients, we discovered that PEDF overexpression in tumor tissues not only connected with higher lipid contents but also directly associated with expressions of key enzymes involved in FFA metabolism (Fig. 4). Our study further revealed that PEDF exacerbated lipogenesis pathway, while impaired FFA oxidation pathway, causing enhanced lipid accumulation in HCC cells (Fig. 5 and Supplementary Fig. 2E, F), which might lead to tumor cell growth. Moreover, depletion of FASN partly reversed the increase in cell proliferation induced by PEDF (Fig. 6a, b), implying FASN as one of the downstream effectors of PEDF regulation on FFA metabolism in HCC.

Early studies focused the lipid regulatory activity of PEDF on its ATGL-binding capability^24,39,40^. However, we previously found PEDF mediate ATGL degradation^41^ and others reported that PEDF mainly bound to laminin receptor in HCC cells and tissues^14^. We observed consistently decreased ATGL expressions in HCC tissues (Fig. 4), which contradicts the increased FFA levels; this could be explained by elevated degradation of ATGL caused by high-expressed PEDF in HCC^41^ and indicates lipolytic pathway mediated by ATGL may not be responsible for the raised FFA levels in HCC.

AMPK, whose activity modulated by phosphorylation, is considered as a crucial energy sensor that regulates cellular metabolism. In the liver, AMPK activation suppresses DNL pathway by blocking FASN transcription and stimulates FFA oxidation through increasing CPT1α levels and subsequently transporting FFA into the mitochondria^28,42^. AMPK and its upstream kinase, Liver Kinase B1, are often regarded as tumor suppressors that expression was reduced in most cancers^43–45^. In line with these findings, we discovered decreased expression and activation of AMPK in tumor tissues of HCC patients. In addition, the activation was inversely associated with PEDF expression (Fig. 4). Further experiments showed that high-expressed PEDF steadily repressed AMPK activation, whereas enhanced FFA levels, both in vitro and in vivo (Fig. 5). Moreover, AMPK-specific agonist, AICAR, could efficiently attenuate the increased lipogenesis pathway, lipid accumulation, and cell growth induced by PEDF overexpression (Fig. 6). Collectively, we proposed AMPK activation might be the main target, which PEDF acts on, to regulate FFA metabolism during HCC development.

In addition to phosphorylation, recent studies have established that AMPK activity is also regulated by ubiquitination^29–31,46^. Polyubiquitination of AMPK subunits can downregulate AMPK activity through proteasomal degradation^29–31,46^. Makorin ring finger protein 1 was recently identified as an E3 ubiquitin ligase for AMPKα in the liver^29^. Consistently, we found PEDF effectively shorten protein half-lives of both AMPK and pAMPK via proteasomal degradation pathway (Fig. 7a, b). Furthermore, ectopically expressed PEDF with MG132 treatment markedly promoted the polyubiquitination levels of AMPK and pAMPK (Fig. 7c, d). These data in combination with the functional findings (Figs. 5 and 6) suggest that PEDF impairs AMPK activation and level through ubiquitin–proteasome-mediated protein degradation. As we were unable to detect direct interaction between PEDF and AMPK (Supplementary Fig. 4D, E), the underlying mechanism how intracellular PEDF modifies AMPK ubiquitination still needs further investigation.

In conclusion, our study first demonstrated that intracellular PEDF restrained the activation of AMPK via UPS, induced lipogenesis pathway, whereas inhibited FFA oxidation pathway, leading to elevated FFA levels and lipid accumulation, and eventually promoted HCC cell proliferation. On the other hand, secreted PEDF suppressed tumor angiogenesis as a traditional anti-angiogenic factor in the late stage of HCC progression, together exhibiting the dual regulatory functions of PEDF during HCC development (Fig. 7e). This study helps to clarify early paradoxical results and form a comprehensive understanding of the diverse role of PEDF in HCC.

## Supplementary information

Supplementary material

Supplemental Figure 1

Supplemental Figure 2

Supplemental Figure 3

Supplemental Figure 4

## Data Availability

All data generated or analyzed during this study are included in this article and its Supplementary Information files.
