# Supplementary material for: The contrary intracellular and extracellular functions of PEDF in HCC development
Source: Cell Death Dis. 2019 Oct 3;10(10):742. doi: 10.1038/s41419-019-1976-4 (PMC6776659; doi:10.1038/s41419-019-1976-4)
Supplement: Supplementary file 1 — Supplementary material [file 41419_2019_1976_MOESM1_ESM.docx]

**Supplements**

| Factors | Patients | PEDF | | *p* Value |
| --- | --- | --- | --- | --- |
|  |  | Low | High |  |
| Gender |  | | | |
| Male | 59 | 14 | 45 | 0.278 |
| Female | 9 | 1 | 8 |  |
| Age |  | | | |
| <50 years | 32 | 7 | 25 | 1 |
| ≥50 years | 36 | 8 | 28 |  |
| HBV infection |  | | | |
| Positive | 54 | 10 | 44 | 0.109 |
| Negative | 11 | 5 | 6 |  |
| Clinical Stage |  | | | |
| Ⅰ-stage | 14 | 1 | 13 | 0.098 |
| Ⅱ-stage | 21 | 3 | 18 |  |
| Ⅲ-stage | 33 | 11 | 22 |  |
| Grade |  | | | |
| Well | 16 | 1 | 15 | 0.121 |
| Modeately | 26 | 6 | 20 |  |
| Poorly | 23 | 8 | 15 |  |
| Recurrence |  | | | |
| Positive | 27 | 6 | 21 | 1 |
| Negative | 17 | 4 | 13 |  |
| Survival Rate |  | | | |
| 1 year |  | 66.60% | 73.90% | 0.747 |
| 3 years |  | 21.40% | 36.40% | 0.356 |
| 5 years |  | 7.70% | 18.40% | 0.662 |

Supplemental Table. 1 Correlation between PEDF expression and clinicopathological features in HCC patients.

Correlation analyses were determined by the Chi-squared tests with indicated number of HCC patients (6 HCC samples with unchanged-expression of PEDF were excluded).

Supplemental Fig. 1 Construction of signaling peptide deletion PEDF (mPEDF) plasmid, and the effect of secreted PEDF.

(A) Construction of signal-peptide-deleted PEDF (mPEDF) and full-length PEDF (PEDF) overexpression plasmids. (B & C) HepG2 cells transfected with control (CON-HepG2), PEDF (PEDF-HepG2), or mPEDF (mPEDF-HepG2) plasmids for 24hr and then starved for 24hr. (B) Culture medium were collected and secreted PEDF were evaluated with ELISA (n=3). (C) Cells were harvested and subjected to Western Blot analysis. (D) Conditioned media (CM) from stable CON-, PEDF- and mPEDF-HepG2 cells were collected to treat Human umbilical vein endothelial cells (HUVECs) for 72hr. The viable cells were quantified by MTT assay (n=5). All data are presented as mean±S.D., and *** denotes p<0.001.

Supplemental Fig. 2 Intracellular PEDF enhances lipid accumulation and cell growth in stable BEL-7402 cells.

Stable CON-, PEDF- and mPEDF- BEL-7402 cells were generated as described in “Materials and Methods”. Cells were harvested and subjected to (A) Western Blot analysis and (B) MTT assay (n=5). (C & D) Colony formation assay was performed as described in “Materials and Methods”. (C) Representative images and (D) colony formation rates are shown (n=3). (E & F) Lipid droplets in indicated stable BEL-7402 cells were treated with 400μM OA for 6hr and then stained with Oil red O. (E) Representative images and (F) quantification of lipid content are shown (n=3). All data are presented as mean±S.D., and * , **, and *** denotes p<0.05, p< 0.01, and p<0.001, respectively.

Supplemental Fig. 3 FASN inhibition on PEDF-overexpressed HepG2 cell proliferation.

Stable CON- and PEDF- HepG2 cells were treated with either DMSO or a specific FASN inhibitor, C75 (5μg/ml), for 24/48/72hr. The viable cells were quantified by MTT assay (mean±S.D., *** p<0.001, n=6).

Supplemental Fig. 4 PEDF indirectly regulates AMPK activity.

(A-C) Stable CON- and PEDF- HepG2 cells were first starved for 12/24hr and then harvested for qPCR analysis (mean±S.D., ** p<0.01, *** p<0.001, n=3). (D-E) PEDF-HepG2 cells were harvested and immunoprecipitated with either normal-IgG (con)/PEDF antibodies (D), or normal-IgG (con)/AMPK antibodies (E). Western Blot analysis was performed and pAMPK/AMPK/PEDF in all the immunoprecipitates and lysates were detected with pAMPK/AMPK/PEDF antibodies.
