# Supplementary figures and images for: The contrary intracellular and extracellular functions of PEDF in HCC development
Source: Cell Death Dis. 2019 Oct 3;10(10):742. doi: 10.1038/s41419-019-1976-4 (PMC6776659; doi:10.1038/s41419-019-1976-4)

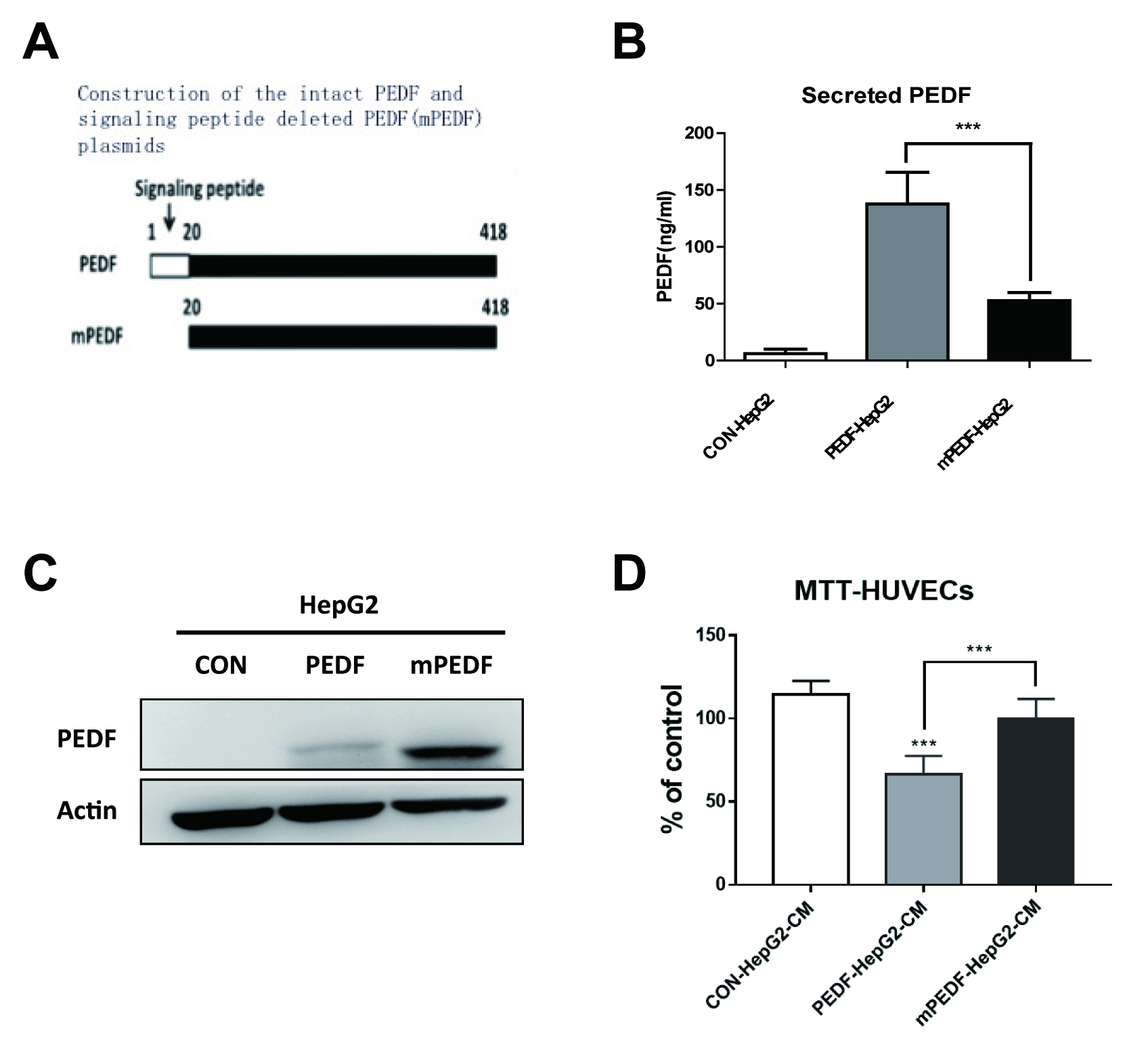

Supplement: Supplementary file 2 — Supplemental Figure 1 [file 41419_2019_1976_MOESM2_ESM.tif]

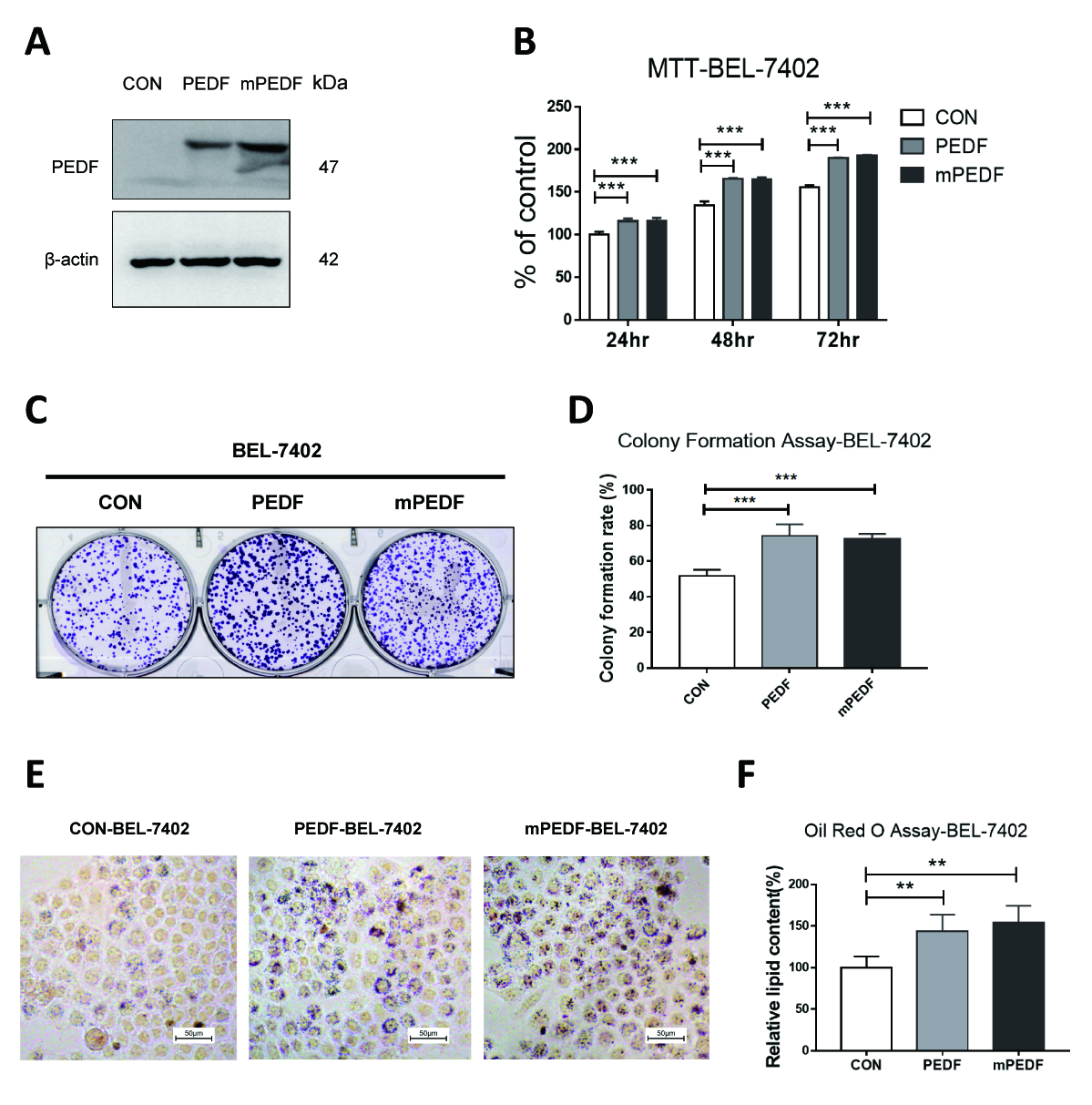

Supplement: Supplementary file 3 — Supplemental Figure 2 [file 41419_2019_1976_MOESM3_ESM.tif]

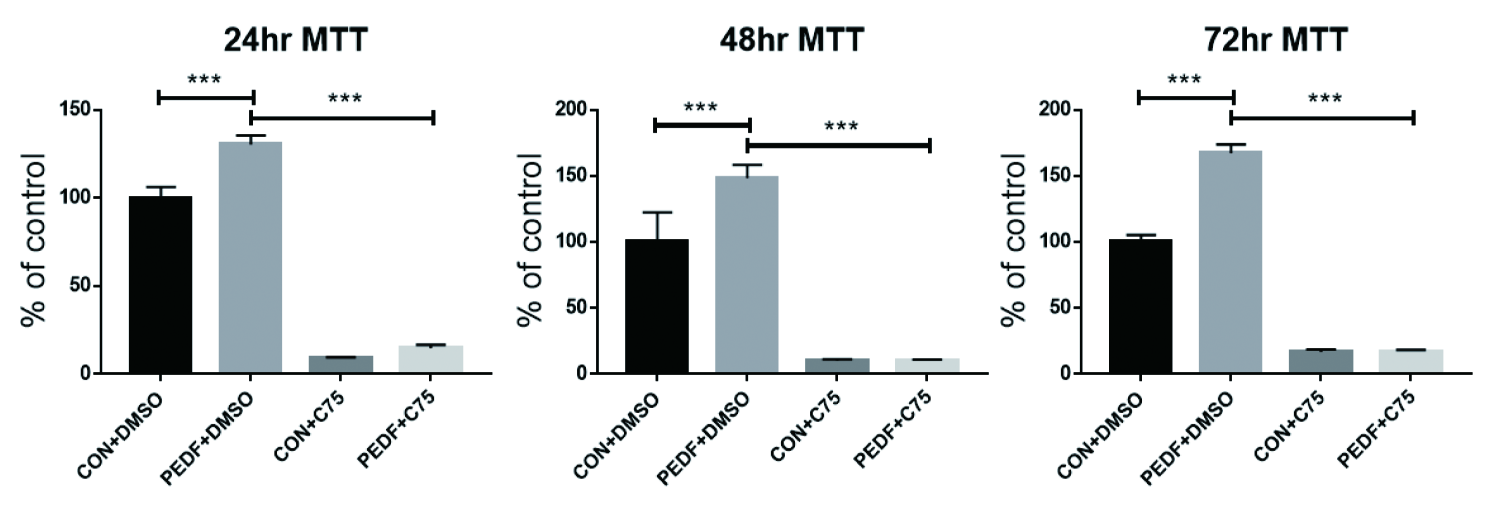

Supplement: Supplementary file 4 — Supplemental Figure 3 [file 41419_2019_1976_MOESM4_ESM.tif]

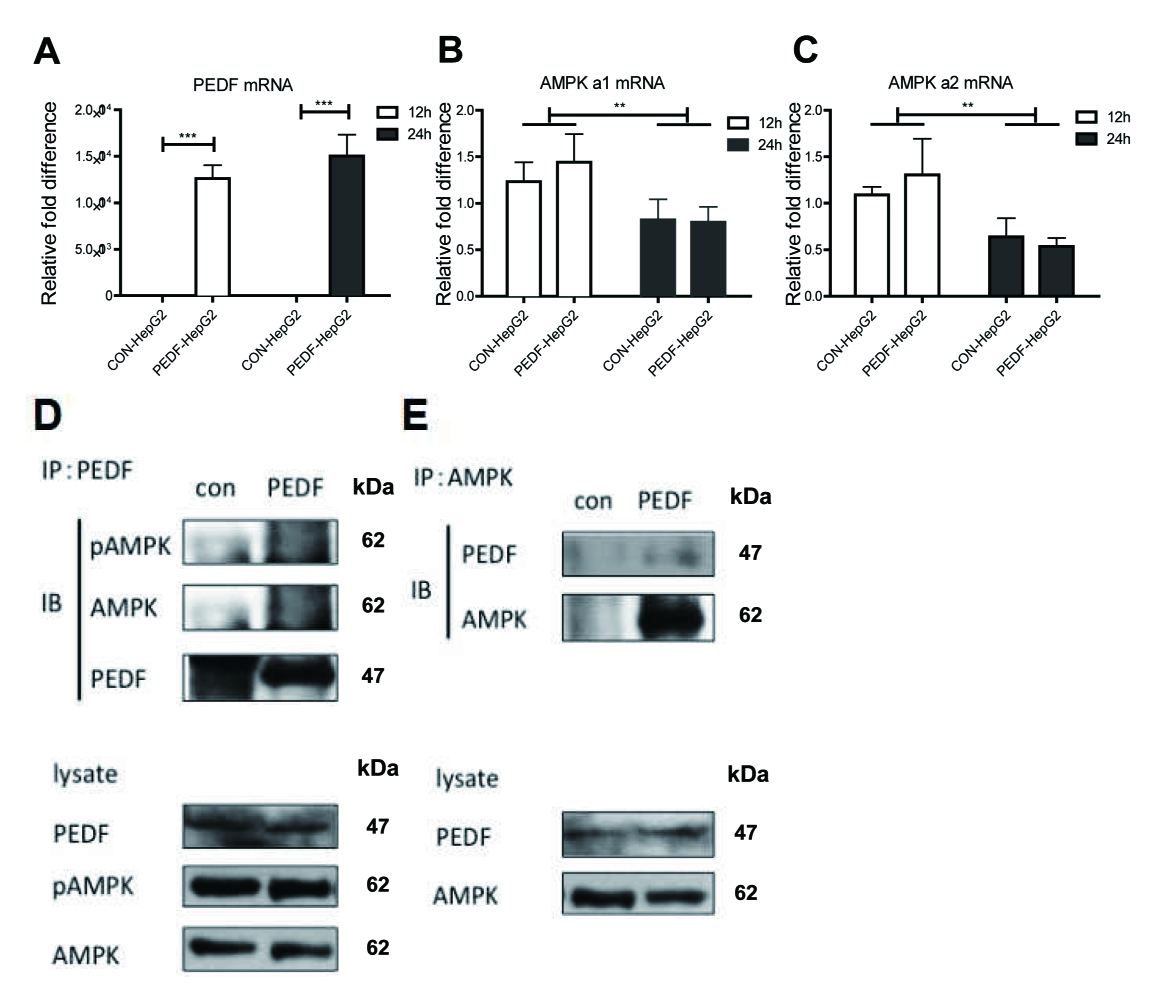

Supplement: Supplementary file 5 — Supplemental Figure 4 [file 41419_2019_1976_MOESM5_ESM.tif]
